# Basic Technology to Support Stimulation Access, Activity, and Correct Posture in Individuals With Intellectual and Multiple Disabilities: Development and Usability Study

**DOI:** 10.2196/92873

**Published:** 2026-08-06

**Authors:** Giulio E Lancioni, Gloria Alberti, Valeria Chiariello, Elia Zacchini, Nirbhay N Singh, Mark F O'Reilly, Jeff Sigafoos, Serafino Buono

**Affiliations:** 1 Lega F. D'Oro Research Center Osimo, Marche Italy; 2 Medical College of Georgia Augusta University Augusta, GA United States; 3 Department of Special Education The University of Texas at Austin Austin, TX United States; 4 Faculty of Education, Health, and Psychological Sciences Victoria University of Wellington Wellington, Wellington Region New Zealand; 5 Oasi Research Institute - Istituto di Ricovero e Cura a Carattere Scientifico Troina, Sicily Italy; 6 Department of Medicine and Surgery Università degli Studi di Enna Kore Enna, Sicily Italy

**Keywords:** blindness, intellectual disability, light sensor, MacroDroid application, motor impairment, pressure sensors, proximity sensor, smartphone

## Abstract

**Background:**

People with severe to profound intellectual disability and visual or visual and motor impairments often experience passivity and isolation. This can lead to limited environmental stimulation, reduced physical activity, and the acquisition of incorrect body postures. Approaches to improve the situation have generally involved increasing environmental stimulation, which was regulated by the context or controlled by the people through adaptive responding and assistive technology.

**Objective:**

This study assessed 2 intervention programs serving as extensions and advancements of previous approaches aimed at helping participants with intellectual and multiple disabilities control environmental stimulation through their own responses and assistive technology. The first program was designed to help 4 participants with severe intellectual disability and blindness alternate between periods of preferred music stimulation and mild physical activity. The second program was designed to help 5 participants with severe to profound intellectual disability, visual and motor impairments, and postural problems (ie, a tendency to tilt their torso and head forward). These participants were led to access preferred environmental stimulation, engage in mild physical activity, and increase correct posture.

**Methods:**

Each of the 2 programs was implemented according to a single-case research design. The technology for the first program involved (1) a smartphone with the MacroDroid app, which regulated the intervention conditions; (2) pressure sensors that allowed the participants to activate music stimulation; (3) a proximity sensor that monitored the participants’ physical activity responses (ie, placing objects in a container high up in front of them); and (4) a mini speaker. The same technology was also used for the second program. In this program, the participants’ physical activity responses consisted of arm-stretching movements, and the smartphone also served for monitoring the participants’ correct posture. Within each program, a session included 7 music periods and 6 activity periods.

**Results:**

During programs 1 and 2, the mean percentage of music pieces activated independently of research assistants’ guidance was between 94.7% and 99.4%. The mean percentage of objects placed in the carton and of shelf-touching responses performed independently was between 98.7% and 99.7%. The baseline mean percentages were between 0% and 24.5%. The mean percentage of session time in a correct posture (program 2) increased to 71.1%-89.6% from baseline values of 18.8%-36.5%. A total of 8 of the 9 participants involved in the 2 programs showed higher levels of indices of happiness during program sessions than control sessions.

**Conclusions:**

The results suggest that the programs might be a useful resource for supporting people with severe to profound intellectual disability and visual or visual and motor impairments.

## Introduction

### Background

People with severe or severe to profound intellectual disability and visual or visual and motor impairments tend to be passive and isolated, with limited or no opportunities to control environmental events (and stimulation) around them [1-5]. Their situation may be largely shaped by their inability to locate, recognize, and operate conventional stimulation sources (eg, television and music devices), their lack of self-determination, their poor motor condition, and a general disinterest in activity engagement [4,6-8]. The negative implications of this situation can include a reduced level of environmental stimulation, limited physical activity, and possibly incorrect body postures, that is, a range of conditions that can further hinder people’s rehabilitation prospects, general well-being, and quality of life [9-15].

The literature available suggests that several stimulation approaches may be potentially useful in curbing, at least partially, the impact of those negative implications. Two common stimulation approaches involve the use of environmental enrichment and multisensory rooms, respectively. Environmental enrichment consists of an increase in the level of environmental stimulation regulated by staff or other personnel in charge of the context [10,14,16-18]. This approach is considered functional for (1) countering the most feared of the negative implications mentioned above, that is, the risk of low stimulation input (with consequent personal impoverishment), and (2) improving the participants’ quality of life [10,19]. Multisensory rooms are structured areas equipped with multiple stimulation sources that are designed to engage the participants’ senses and thus enrich their sensory input and possibly provide them with a feeling of satisfaction [20-23].

A third stimulation approach is characterized by the fact that the stimulation is mediated by direct staff and caregiver involvement (intervention). One strategy used within this approach includes an increase in social (verbal and physical) contact between participants and personnel sharing the same context [24-27]. Another strategy exposes the participants to simple stories whose narration by staff is accompanied by the presentation of stimulation events that underline the content of the stories [28-31].

A fourth stimulation approach, contrary to those mentioned above, ensures that the stimulation is controlled by the participants through their own responses and the use of microswitch (sensor) technology [11,13,32,33]. Specifically, the participants are taught to activate a microswitch device through a specific response (eg, a simple hand movement or a hand-stretching movement involving the use of objects). Such a response becomes instrumental in enabling them to access brief periods of preferred stimulation. The distinctive aspects of this approach are that the participants (1) are actively involved in seeking stimulation (rather than being automatically exposed to it) and (2) are required to increase their physical engagement (ie, through their microswitch activation responses) and thus counter their passivity and detachment.

A final stimulation approach differs from the fourth in that its goal is not limited to bringing the participants to seek environmental stimulation through their own responses but also aims to promote their postural control [34]. For example, participants may be (1) taught to perform specific (functional) arm and hand responses to activate microswitch devices and access preferred stimulation and (2) required to maintain a correct posture during the stimulation to ensure that this continues without interruptions [34,35].

In light of the above, one might argue that the last 2 approaches can be considered advantageous because they allow the participants to have an active role in seeking environmental stimulation and because they promote the participants’ physical engagement and possibly postural improvement. In essence, these approaches can be viewed as valuable complements to standard occupational therapy and physiotherapy within daily contexts [4,9,12]. With regard to this point, it may also be noted that these approaches rely on the use of basic technology and are only marginally demanding on staff time [4,36,37].

### Objectives

The present study was aimed at assessing 2 intervention programs serving as extensions and advancements of the last 2 approaches described above. The first program was directed at 4 participants who presented with severe intellectual disability and blindness. These participants were led to alternate periods in which they could activate preferred music stimulation pieces with periods of mild physical engagement in which a functional response requiring some physical effort was to be repeated several times. Each music stimulation piece lasted 1 minute. Each physical engagement period required the participants to place 6 to 10 objects (ie, common and age-appropriate objects) in a tall carton, which was in front of them. The performance of every response involved forward and upward arm and shoulder stretching (ie, movements that physiotherapists had recommended as useful and appropriate for the participants).

The second program was directed at 5 participants who presented with severe to profound intellectual disability, visual and motor impairments, and postural problems (ie, a tendency to tilt their torso and head forward). These participants were led to alternate stimulation periods such as those described for the previous group of participants with physical engagement periods. During the latter periods, the participants were required to perform upward and sideward arm-stretching responses (ie, responses that physiotherapists considered useful because of the nature of the movements involved and because they were antagonistic to the participants’ postural problems). The participants’ posture was monitored throughout the sessions. Loss of correct posture during a stimulation period made the stimulation inaudible until the correct posture was regained.

The programs were developed by the authors, who have long experience working with and developing technology-aided solutions for people with intellectual and multiple disabilities. The authors operated in continuity with previous research work in the area, as indicated above, and in collaboration with staff personnel and caregivers. Each of the 2 programs was implemented according to a single-case research design. The technology used for the programs included commercial components such as a smartphone, pressure and proximity sensors, an interface to connect the sensors to the smartphone, the MacroDroid app, and a mini speaker.

## Methods

### Ethical Considerations

Staff considered the participants’ involvement in the study to be a beneficial and pleasant experience for them (ie, an experience that extended their daily rehabilitation and care treatment by promoting self-determination, enriching stimulation input, and facilitating physical activity). The participants’ condition did not allow staff to ask them for or obtain their clear consent to be involved in the study. In light of this, their legal representatives read and signed a consent form on their behalf. The consent form specified that there was no compensation for the participants’ involvement, that participants could be withdrawn from the study at any time, and that their data would be deidentified. The study was approved by the Ethics Committee of the Lega F. D’Oro, Osimo (AN), Italy (P031020252). All procedures applied during the study were in line with internationally recognized ethical standards and complied with the 1964 Helsinki Declaration and its later amendments.

### Participants

Table 1 reports the chronological age, Vineland age equivalents for daily living skills (personal subdomain), and sensory-motor impairments for the 9 participants. The first 4 (identified with the pseudonyms of Eloise, Grace, Jackson, and Margot) were involved in program 1, and the last 5 (identified with the pseudonyms of Matias, Colton, Alina, Hazel, and Graham) were involved in program 2. Their chronological age ranged from 27 to 59 years. The Vineland age equivalents for daily living skills (personal subdomain) measured via the second edition of the Vineland Adaptive Behavior Scales [38,39] varied between 1 year and 7 months and 4 years. The participants had a diagnosis of congenital encephalopathy and intellectual disability, which was combined with blindness or severe visual impairment. Four of the last 5 participants also presented with motor impairments that precluded any ambulation or allowed only limited levels of supported ambulation. While no recent IQ scores were available, the psychological records of the rehabilitation and care centers that the participants attended reported the participants’ level of intellectual disability to be in the severe range (first 4 participants) or the severe-to-profound range (last 5 participants).

The participants were included in the study based on a number of conditions, which had been preliminarily verified. First, they tended to be passive and dependent on staff to access environmental stimulation and engage in physical activity (eg, using objects or performing functional body movements). Second, they seemed to enjoy different types of music stimulation. Third, they were considered capable of responding to simple verbal instructions concerning basic actions such as touching or putting away objects. Fourth, staff were favorable to the programs and the technology involved, which had been illustrated to them in advance.

**Table 1:** Participants’ chronological age, Vineland age equivalents for daily living skills (personal subdomain), and sensory-motor impairments.

Participant (pseudonym)	Chronological age (years)	Vineland age equivalents^a^, years (months)	Sensory-motor impairments
Eloise	59	3 (10)	Blindness
Grace	41	2 (7)	Blindness
Jackson	35	3 (3)	Blindness
Margot	50	4 (0)	Low vision
Matias	58	2 (4)	Minimal residual vision Lack of ambulation
Colton	52	2 (8)	Blindness Lack of ambulation
Alina	27	3 (1)	Blindness
Hazel	45	2 (4)	Minimal residual vision Lack of ambulation
Graham	48	1 (7)	Blindness Lack of ambulation

^a^Age equivalents are based on the Italian standardization of the Vineland Scales [38].

### Setting, Sessions, Research Assistants, and Music Stimulation

A quiet room in the participants’ daily contexts, fitted with a desk at which the participants sat, served as the setting for the baseline and the program sessions (see below). The participants’ regular occupational room served as the setting for the control sessions (see below). Sessions were typically carried out once per day, 5 or 6 days a week. A total of 4 research assistants implemented the sessions. They had a university education and were experienced in applying technology-aided programs with people with extensive disabilities and using various data recording procedures.

Different music pieces (songs and other music stimuli) recommended by staff were evaluated to determine whether they would be used during the study. The evaluation was carried out via a stimulus preference screening procedure. The procedure required that 3 brief segments of each music piece suggested by staff for the individual participants be presented nonconsecutively (at least 10 times per segment) over different screening occasions. Only the music pieces whose segments were considered (ie, by the research assistant and staff member involved in the screening procedure) to produce positive reactions (eg, alerting and smiling) in at least 50% of their presentations were retained for use in the study [4,13].

### Technology System: Components

**Figure 1:**
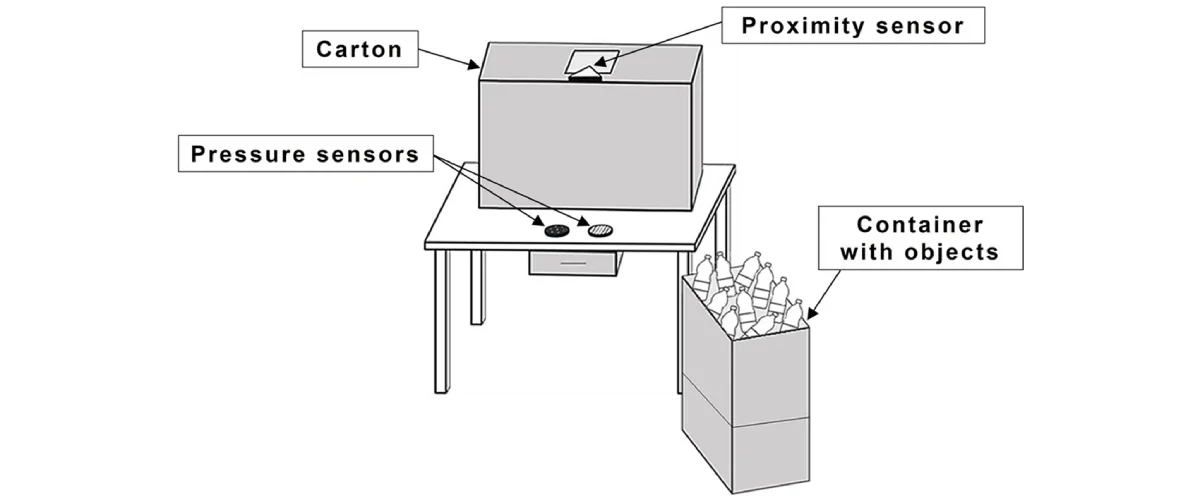
Schematic representation of the 2 pressure sensors placed on the desk, the proximity sensor fixed to the carton, and the container with objects.

**Figure 2:**
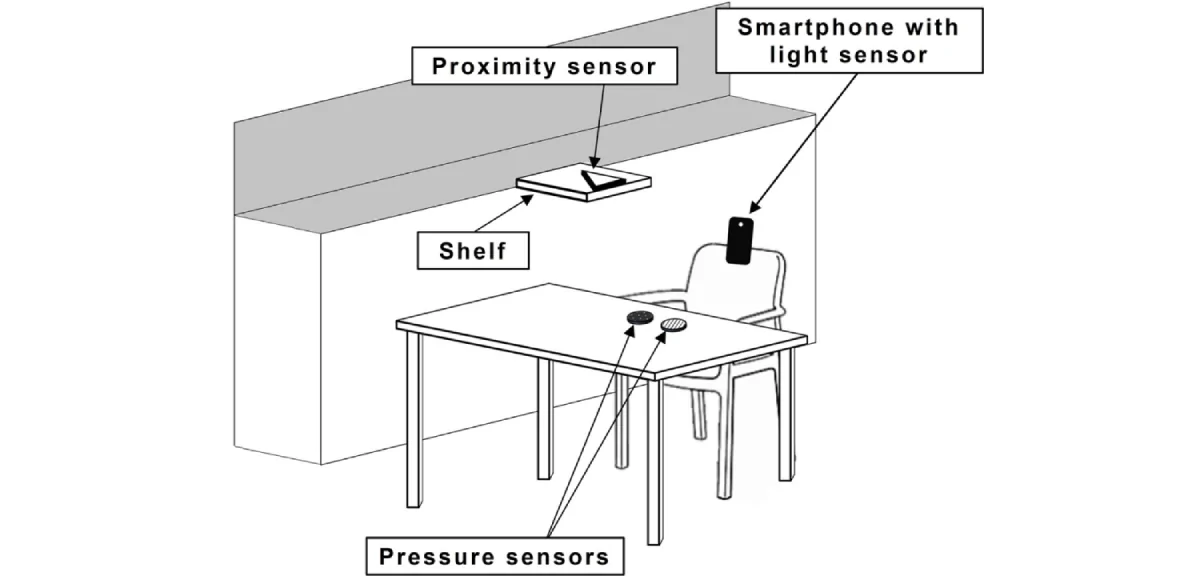
Schematic representation of the 2 pressure sensors placed on the desk, the proximity sensor fixed to the shelf, and the smartphone with the light sensor fixed to the back of the chair.

The technology system used for the programs involved (1) a smartphone operating on Android and fitted with a light sensor, (2) a Bluetooth Encore Plus interface (leonardoausili.com), which was linked to 2 pressure sensors and a proximity sensor, and (3) a Bluetooth mini speaker (JBL Go 3) amplifying the smartphone’s volume. The smartphone was connected to the internet and fitted with the Encore Plus and MacroDroid apps. The pressure sensors consisted of small Buddy buttons, circles with a diameter of 6.3 cm (leonardoausili.com). The proximity sensor consisted of a Big Candy Corn, a triangle with a side of about 10 cm (leonardoausili.com). In program 1, the pressure sensors were on the desk while the proximity sensor was fixed to the carton in which the participants were to place common, age-appropriate objects taken from a container (Figure 1). The smartphone was in the desk drawer. In program 2, the pressure sensors were on the desk, the proximity sensor was on a small shelf that the participants were to touch, and the smartphone was attached to the back of their chair (Figure 2) or to their wheelchair’s headrest.

In program 2, the smartphone on the back of the chair or wheelchair’s headrest served to record whether the participants’ torso or head posture was correct or incorrect. The closer the participant’s torso or head was to the smartphone’s light sensor, the lower the level of light reaching the sensor. Based on this relationship, the smartphone was programmed to record the posture as correct up to a prefixed level of light reaching the sensor. Beyond that level, the posture was considered incorrect. This smartphone and light sensor functioning was successfully tested prior to the study by 2 judges (a staff member and a research assistant), who compared the smartphone’s (correct or incorrect) posture recording with video images of the posture collected simultaneously. Testing was conducted for each participant under environmental illumination conditions that remained stable during the baseline and program sessions.

### Technology System: Functioning

**Figure 3:**
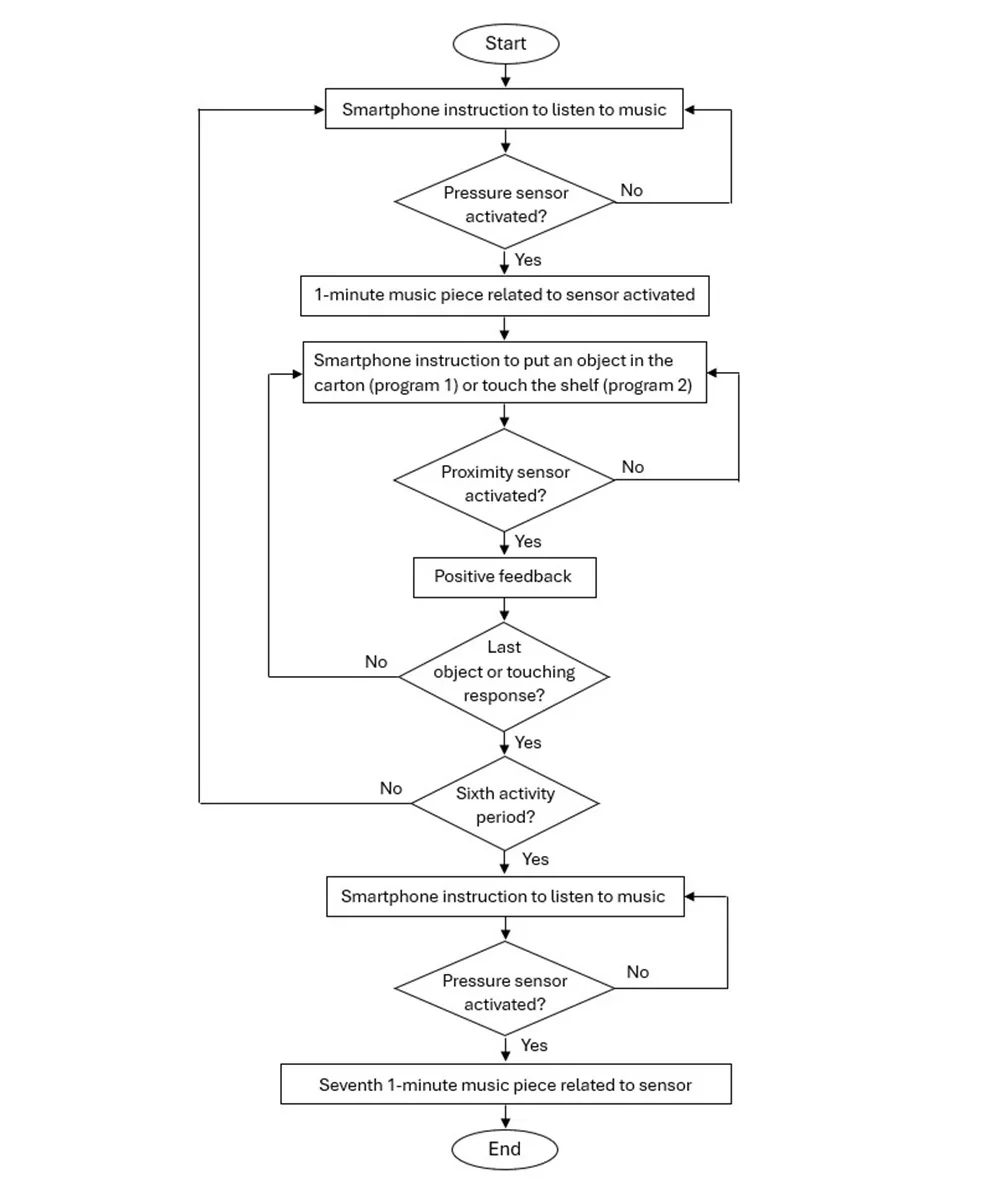
Flowchart summarizing the system’s functioning.

A session with either program started with the smartphone instructing the participants (via the mini speaker) to listen to music (eg, to select the name of a singer or the name of a band). Figure 3 provides a summary of the system’s functioning. The participants could touch (activate) one of the pressure (music) sensors available in front of them, thus sending a signal to the smartphone. The smartphone, using the Encore Plus and the MacroDroid apps, (1) detected and discriminated the signal and (2) presented, via the mini speaker, a 1-minute music piece consistent with the sensor activated. In program 2, the piece was audible to the participants if their posture (as determined by the smartphone fixed on the back of their chair or headrest) was correct and inaudible if their posture was incorrect. In the latter case, the smartphone instructed them to straighten their body or raise their head. As the participants regained the correct posture, the music piece became audible again.

Following the end of the music piece, an activity period started during which the music sensors were unresponsive. The smartphone instructed the participants to put an object in the carton (program 1) or touch the shelf (program 2). These responses activated the proximity sensor fixed near the carton’s opening (program 1) or on the shelf (program 2). Proximity sensor activation led the smartphone (supported by the Encore Plus and the MacroDroid apps) to (1) count each response (ie, an object placed in the carton or a shelf-touching response), (2) provide a one-word positive feedback statement for each response, (3) follow each feedback statement with the instruction to take another object (program 1) or touch the shelf again (program 2), and (4) determine when the participants had produced the number of responses programmed for the activity period (ie, had placed 6-10 objects in the carton or touched the shelf 6-9 times). Once the programmed number was reached, the smartphone instructed the participants to listen to music (ie, as above) and made the proximity sensor on the carton or shelf unresponsive. The instruction to listen to music or to place an object in the carton or touch the shelf was repeated at intervals of 10 seconds until the participants responded to it. The session continued with new opportunities to access preferred music pieces followed by activity periods according to the conditions described above. A session was programmed to include 7 music pieces and 6 activity periods. During the 6 activity periods, the participants were expected to place a total of 36 to 60 objects in the carton or to touch the shelf a total of 36 to 54 times.

### Measures and Data Recording

The four measures recorded during both programs involved (1) the number of music pieces activated independently of research assistants’ guidance, (2) the number of objects placed in the carton or of shelf-touching responses performed independently of research assistants’ guidance, (3) the length of the sessions, and (4) the participants’ indices of happiness (eg, behaviors such as alerting or orienting to the music, smiling, and making music-based rhythmic movements [4,11,40-43]). The fifth measure, recorded only during program 2, concerned the amount of session time the participants spent in a correct posture. With the exception of indices of happiness, the measures were also recorded during the baseline phase that preceded the programs. Indices of happiness were recorded during 25-30 program sessions and as many control sessions (see below).

The first 2 measures were recorded by the research assistants during the baseline and by the technology system during the programs. During the programs, the system’s recorded number of music pieces activated, of objects placed in the carton, and of shelf-touching responses performed would be modified (reduced) if research assistants’ guidance had occurred in the process. The length of the sessions and the time spent in a correct posture were recorded by the technology system throughout the study. The indices of happiness were recorded from videos of the sessions using a partial-interval strategy in which 10-second intervals were used for observing and 5-second intervals were used for recording [4].

Interrater agreement was assessed over (1) all baseline sessions for the first 2 measures and (2) 50% of the program and control sessions for indices of happiness with the involvement of a reliability observer. The percentage of agreement on the first 2 measures (computed by dividing the number of sessions in which research assistants and the reliability observer had scores differing by no more than 1 unit by the total number of sessions and multiplying by 100%) was 100% for all participants. The percentage of agreement for indices of happiness (computed for the single sessions by dividing the number of intervals with agreement by the total number of intervals and multiplying by 100%) ranged between 87.5% and 100%, with means exceeding 93% for all participants.

### Experimental Conditions and Data Analysis

#### Overview

The first 4 participants (ie, the group involved in program 1) and the last 5 participants (ie, the group involved in program 2) were initially exposed to a baseline phase without the technology system. Then, they entered the scheduled program during which the technology system described above was used. For each of the 2 groups, the program was implemented according to a nonconcurrent multiple-baseline design across participants (ie, the program started after the group members had received different numbers of baseline sessions) [44,45]. Parallel to 25-30 program sessions, both groups received control sessions. The latter sessions, which were implemented immediately before the program sessions, served to determine whether the program sessions had an impact on the participants’ indices of happiness [4,46].

Two basic strategies were used to ensure high levels of accuracy in the research assistants’ implementation of the sessions (ie, high levels of procedural fidelity [47,48]). First, the research assistants were involved in preliminary training through simulated sessions in which they were to apply the procedural conditions. Such training continued until they had shown the ability to manage those conditions. Second, the research assistants were provided with feedback about their performance during the study. The feedback (which they received from a study coordinator who had access to videos of the sessions) consisted of being informed as to whether their performance was accurate or included inaccuracies that needed to be amended (with the latter being virtually absent and thus confirming the research assistants’ procedural correctness).

The Nonoverlap of All Pairs (NAP) method (ie, a method for the analysis of single-case research data [49,50]) was used to assess the effect size (1) of program 1 on the number of music pieces activated, objects placed in the carton, and indices of happiness, and (2) of program 2 on the number of music pieces activated, shelf-touching responses performed, session time spent in a correct posture, and indices of happiness displayed.

#### Baseline Sessions

The baseline phase included 5-10 sessions for the first 4 participants (who received program 1) and 5-11 sessions for the last 5 participants (who received program 2). The first four participants sat at a desk with (1) a smartphone that could be used for accessing preferred songs, (2) a carton placed in front of them, and (3) a container with objects placed to their right. The last five participants had (1) a smartphone that could be used for accessing preferred songs; (2) a smartphone with a light sensor and the MacroDroid app, fixed on the back of their chair or their wheelchair’s headrest (Figure 2), which served to record the session time spent in a correct posture; and (3) a small shelf with a proximity sensor on it, which was fixed high up to their right.

The smartphone that the participants could use was set up to play music pieces from a YouTube playlist. The playlist had been preliminarily arranged for the individual participants based on their preferred music pieces. The playlist was regulated through a play and pause button on the smartphone’s screen. To mark the button area for the participants (given their visual impairment), a small cotton ball was placed just below that area. Touching the smartphone screen above the cotton ball (ie, activating the play and pause button) would start or stop the music.

At the beginning of a baseline session, the research assistants helped the first four participants (1) touch the smartphone, locate the cotton ball, and activate the music briefly; (2) touch the carton’s opening; and (3) take an object from the container and place it in the carton. The difference for the last 5 participants was that they were helped to touch the shelf, rather than to touch the carton and place an object in it.

After this introductory help, the research assistants instructed (encouraged) the participants to proceed on their own. If the participants managed to activate a music piece, place objects in the carton, or touch the shelf on their own, their responses were recorded as independent of research assistants’ guidance. If the participants did not respond to the instruction (encouragement) for 25 seconds, the research assistants provided them with guidance (ie, verbal and/or physical support) to activate a music piece, which was then recorded as dependent on guidance. Any music piece was interrupted after 1 minute. If the participants remained passive for 25 seconds following the end of the music piece, the research assistants provided them with guidance to put an object in the carton (first 4 participants) or touch the shelf (last 5 participants). The session continued until (1) the participants had activated 7 music pieces and had placed the objects in the carton or performed the shelf-touching responses programmed for them or (2) 4 consecutive instances of research assistants’ guidance had occurred.

#### Program Sessions

Programs 1 and 2 included 59-73 and 61-80 sessions (with differences due to participants’ availability), respectively. The participants were provided with the technology system, which worked as described in the Technology System: Components and Technology System: Functioning sections and Figure 3. The programs were introduced by 2 practice sessions during which the research assistants could repeat the smartphone’s instructions and help the participants respond to those instructions. During the following sessions, the research assistants would provide verbal and physical guidance if the participants did not respond to a smartphone instruction after this had been presented 3 times. During 25-30 sessions spread over the central and final part of the programs, data recording also involved the participants’ indices of happiness (eg, behaviors such as alerting or orienting to the music, making music-based rhythmic movements, smiling, or vocalizing [40-43]). Recording occurred within the first 10 minutes of the sessions so that the data could be compared to those of 10-minute control sessions.

#### Control Sessions

Each of the 25-30 program sessions in which indices of happiness were recorded was preceded by a 10-minute control session involving the recording of the same indices. During the control sessions, the participants were not involved in specific activities but had objects available to them and could have some interaction with staff and listen to staff interacting with other individuals with intellectual and visual disabilities sharing the same context. Recording the participants’ indices of happiness during program sessions and parallel control sessions served to determine whether the programs had an impact on those indices.

## Results

**Figure 4:**
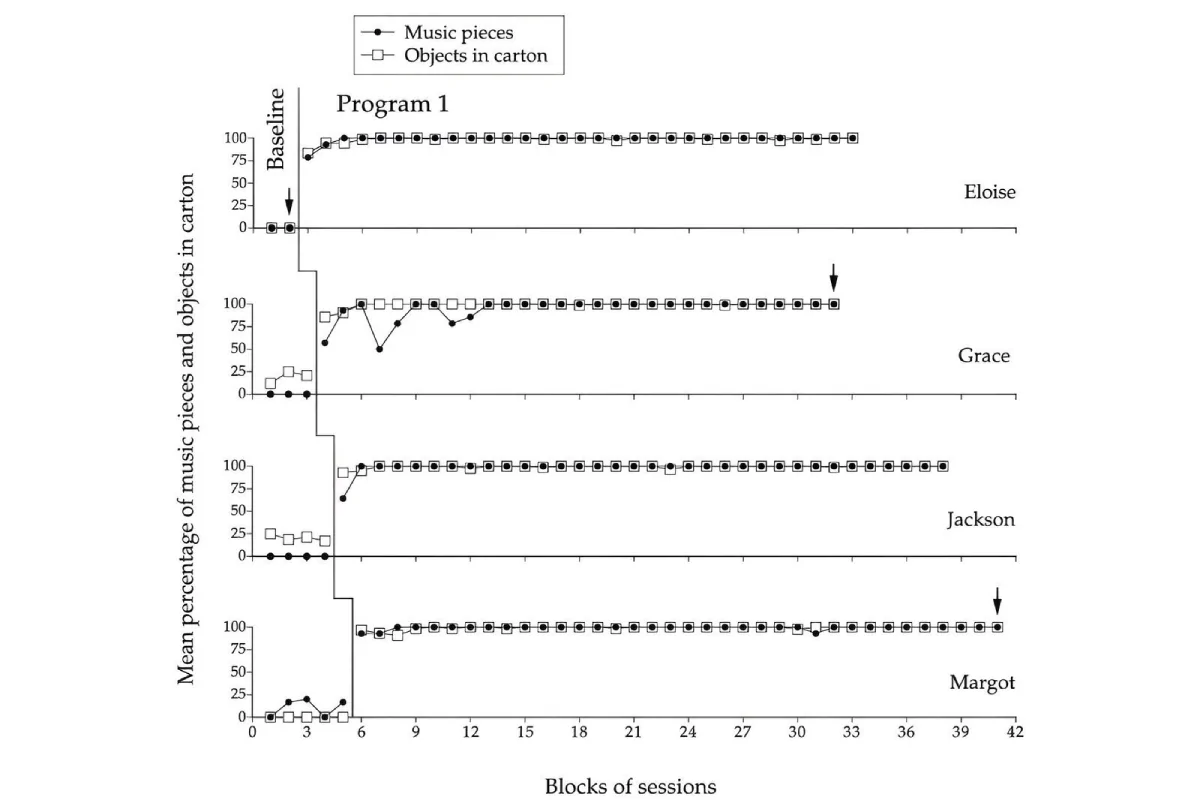
Data for the 4 participants involved in program 1. Dots and open squares represent the mean percentage of music pieces activated and objects placed in the carton independently of research assistants’ guidance, respectively, over 2-session blocks. An arrow indicates 3-session blocks.

**Figure 5:**
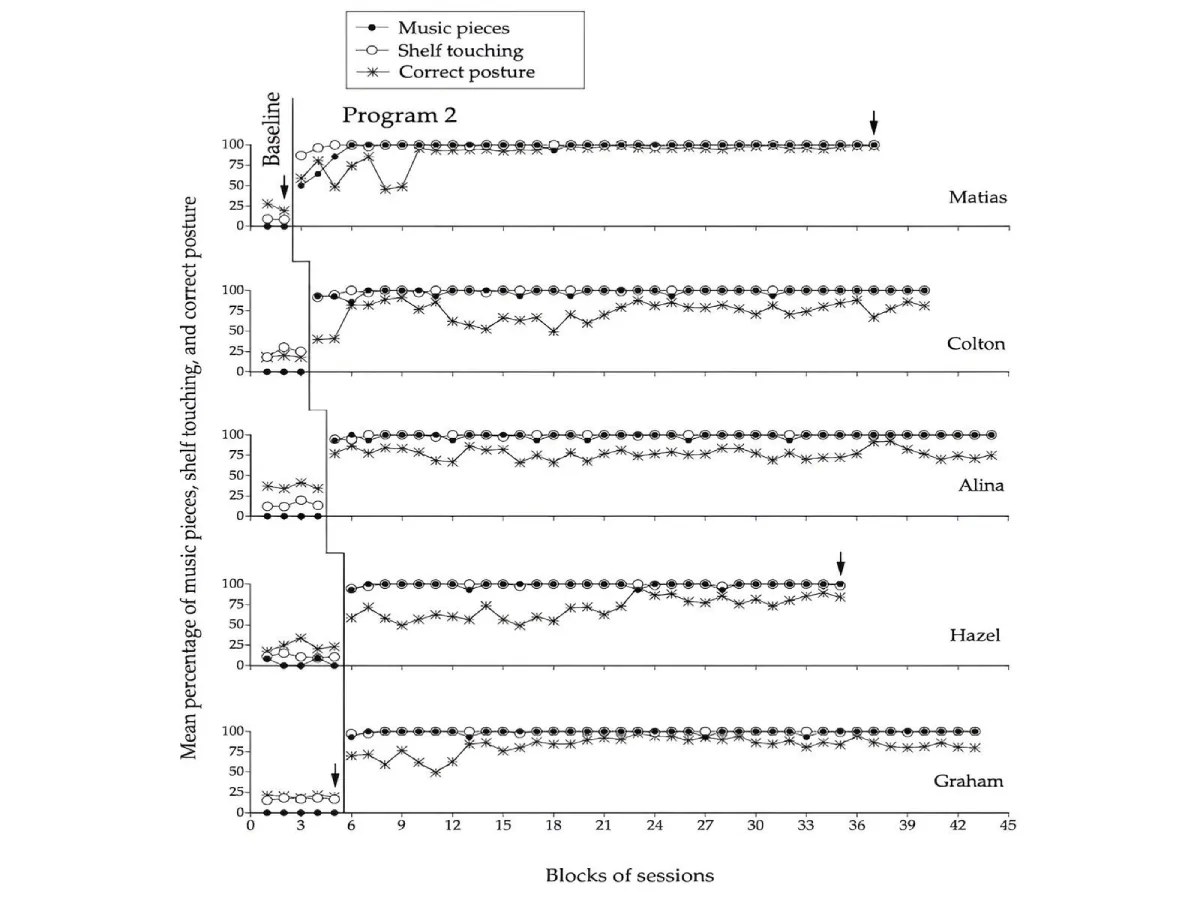
Data for the 5 participants involved in program 2. Dots, open circles, and asterisks represent the mean percentage of music pieces activated, shelf-touching responses performed independently of research assistants’ guidance, and session time spent in a correct posture, respectively. Data are plotted as in Figure 4.

Figure 4 reports the data for the first 4 participants on music pieces activated and objects placed in the carton independently during program 1 and the preceding baseline sessions. Figure 5 reports the data for the last 5 participants on music pieces activated and shelf-touching responses performed independently and for session time spent in a correct posture during program 2 and the preceding baseline sessions. The data points appearing in the figures are mean percentage values computed over blocks of 2 sessions (blocks of 3 sessions are marked with an arrow). Percentage values were computed over (1) the music pieces played, (2) the objects used or shelf-touching responses completed during the activity periods, and (3) the session durations (ie, for correct posture). During the program sessions (which were never interrupted), 7 music pieces were played and 6 activity periods were regularly completed (involving totals of 36-60 objects or 36-54 shelf-touching responses). During the baseline sessions (which were always interrupted), the music pieces played and activity periods started could vary between 2 and 6 (with variable numbers of response opportunities).

During the baseline sessions preceding the introduction of programs 1 and 2, the mean percentage of music pieces activated independently of research assistants’ guidance was 0% except for Margot and Hazel. Their mean percentage was 10.7% and 3.5%, respectively. The mean percentage of objects placed in the carton (Figure 4) varied between 0% (Eloise and Margot) and 20.5% (Jackson), with the values of the last 2 or 3 sessions never exceeding the highest values reached in the phase. The mean percentage of shelf-touching responses (Figure 5) ranged between 8.7% (Matias) and 24.5% (Colton). The mean percentage of session time spent in a correct posture (Figure 5) was between about 18.8% (Colton) and 36.5% (Alina). All baseline sessions were interrupted after 4 consecutive instances of research assistants’ guidance. Their mean length was always below 9 minutes.

During programs 1 and 2, the mean percentage of music pieces activated independently of research assistants’ guidance was between 97% and 99.4%, except in the case of Grace, whose level was 94.7%. The mean percentage of objects placed in the carton and shelf-touching responses performed independently was between 98.7% and 99.7%. It may be noted that the lower percentage values were basically concentrated in the initial sessions of the programs, except in the case of Grace (Figure 4). Her lower values for music pieces activated independently spread beyond the initial sessions as she (1) could continue placing objects in the carton without responding to the smartphone’s instruction to listen to music and thus (2) would receive research assistants’ guidance. The mean percentage of session time spent in a correct posture (Figure 5) increased to between about 71.1% (Hazel) and 89.6% (Matias). The mean length of the sessions for the 9 participants ranged between about 12 minutes (Jackson) and 16 minutes (Colton and Hazel).

The NAP method used to compare the program and baseline data on the aforementioned measures showed indices of 1 (as the program data on each measure showed percentage values higher than the baseline values for all participants). These indices, together with the fact that the change from the baseline to the program sessions was rapid and clearly visible, may be taken to confirm the large effect size of the programs.

**Figure 6:**
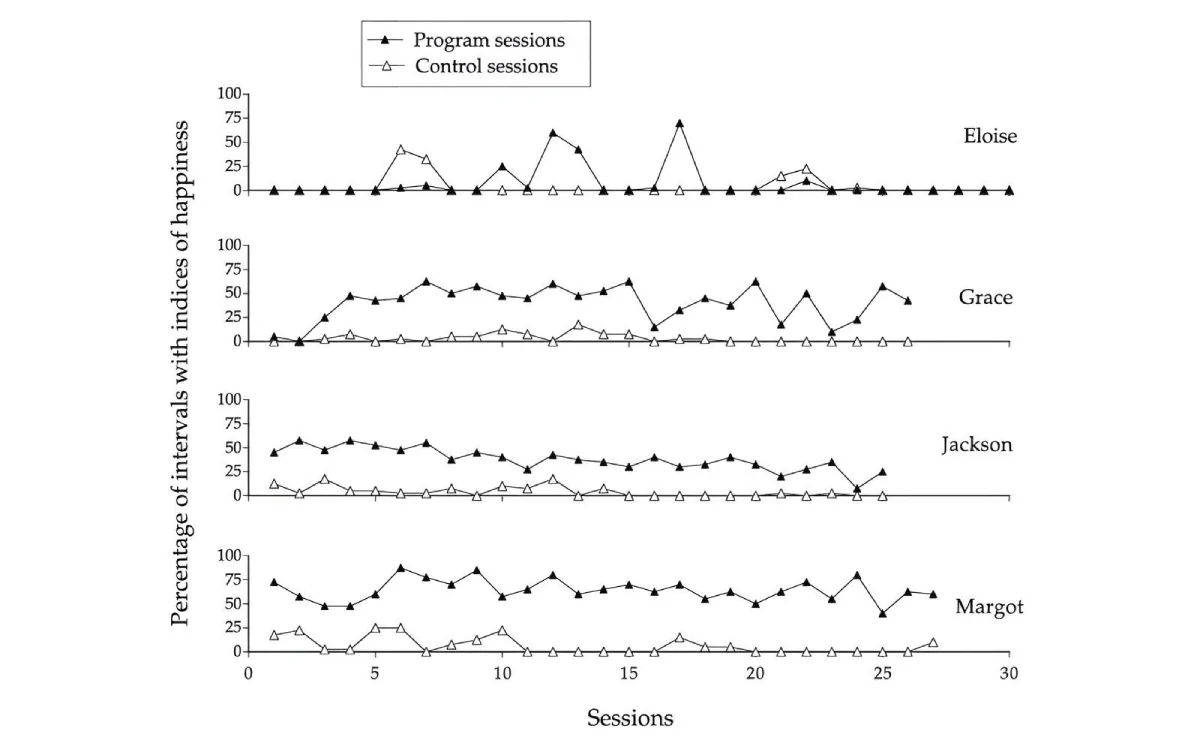
Data for the 4 participants involved in program 1. Black and open triangles indicate the percentage of intervals with indices of happiness during program and control sessions, respectively.

**Figure 7:**
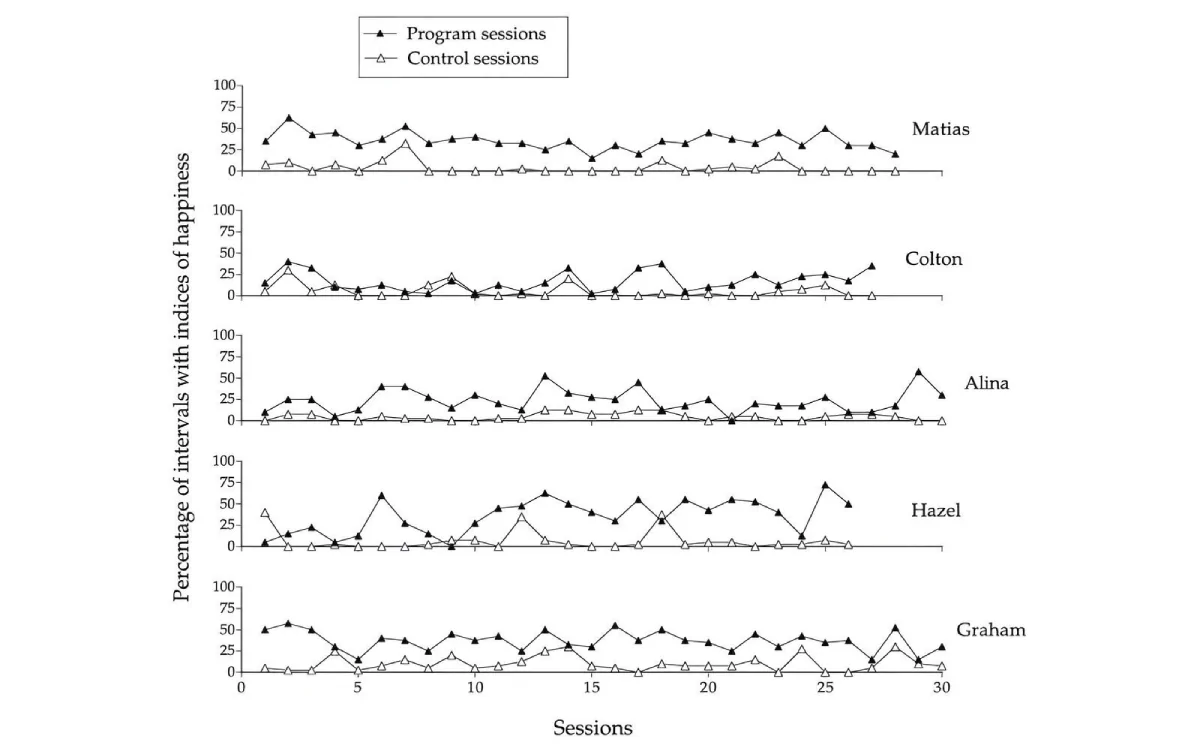
Data for the 5 participants involved in program 2. Black and open triangles indicate the percentage of intervals with indices of happiness during program and control sessions, respectively.

Figures 6 and 7 report the percentage of intervals with indices of happiness during program and control sessions for the first 4 participants and the last 5 participants, respectively. The mean percentage ranged from 7.3% (Eloise) to 64.3% (Margot) during the sessions of program 1 and from 16.8% (Colton) to 37% (Graham) during the sessions of program 2. The mean percentage during the control sessions was below 10% except for Graham (whose mean percentage was 10.2%). The NAP method used to compare the data from the program sessions with the data from the control sessions showed that the programs had a strong effect (with indices of 0.93 to 1) for Jackson, Margot, Matias, Alina, and Graham, a moderate effect (with indices of 0.82 and 0.90) for Colton and Hazel, and no effect (with an index of 0.56) for Eloise.

## Discussion

### Principal Findings

The findings suggest that the programs assessed in this study were helpful in improving the situation of people with intellectual disability and visual or visual and motor impairments. All participants learned to activate music stimulation and to engage in recommended forms of mild physical activity. The participants involved in program 2 also showed an improvement in body posture. Taken as a whole, these findings seem to confirm and extend previous data in the area [33,35,51,52] and to provide new encouraging evidence about the possibility of developing technology-aided programs to help participants with extensive disabilities become responsible for increasing their stimulation input and enhancing their physical activity and postural control. In light of these findings, several considerations are in order.

First, helping people with extensive disabilities manage their access to environmental stimulation and the performance of functional physical actions may be considered relevant from a rehabilitation standpoint and from a practical standpoint. Regarding the rehabilitation aspect, one can argue that the participants’ behavioral activation and self-determination not only curb the risks of stimulation impoverishment and limited physical activity but also likely provide a basis for the participants’ developmental progress, well-being, and satisfaction [15,53-57]. Regarding the practical aspect, one may consider the findings a demonstration that relevant participants’ achievements can be ensured (1) through the support of rather simple and easily accessible technology and (2) with relatively small staff time investment [3,9,36,58-60].

Second, interspersing physical activity with access to preferred music stimulation was thought to be a useful strategy to (1) deal with the participants’ low level of interest in and tendency to avoid such activity (ie, a generally nonpreferred type of engagement) and (2) create a link between the activity and the following preferred stimulation with the likelihood that this would become a reinforcing (motivating) factor for the participants’ activity engagement [61-64]. Pursuing an improvement in body posture could be considered a useful supplement to the goal of increasing physical activity in participants with motor impairments and postural problems. Bringing participants to control their posture through motivational strategies may be the only way to ensure the independence of such control from external supervision [62,64-67]. The aforementioned hypotheses on promoting physical activity and postural control seem to find support in the participants’ performance during the 2 programs. Interpretation of the data reported on postural control may require some caution as the sensor (ie, the smartphone’s light sensor) used to record body posture is not a conventional (validated) instrument for use in that specific assessment area.

Third, while the importance of increasing physical activity in people with extensive disabilities is largely acknowledged [9,10,36,37,51,58-60], the evaluation of the beneficial effects of such an increase is difficult or practically impossible when the activity is mild and lasts for relatively short periods of time. In this case, in fact, one cannot rely on any specific assessment criteria and tools. A similar consideration can be extended to the issue of postural control. Identification of functional assessment criteria and tools may be viewed as an important research objective in this area.

Fourth, the data on indices of happiness show that 8 of the participants found their involvement in the program sessions more satisfactory (enjoyable) than their involvement in the control sessions (ie, sessions featuring common conditions in periods of no specific activity or direct supervision). This evidence, together with the findings that all participants had high and stable levels of performance throughout the program sessions, suggests that (1) the program conditions were largely motivating, (2) the positive impact of the stimulation available in the program sessions exceeded the cost (efforts) of the physical activity or postural control pursued within those sessions, and (3) technology-aided programs such as those reported might help promote participants’ initiative and functional occupational engagement and foster their developmental progress, with possibly positive implications for their quality of life [4,33,37,40,43,57,61,63].

Fifth, the components of the technology system used in the programs, that is, the smartphone, the Encore Plus interface and app, the pressure sensors, the proximity sensor, the mini speaker, and the MacroDroid app, are commercially available and easily affordable. Commercial availability and affordability make the acquisition of the technology and the application of the programs reported in this study feasible for daily contexts [68,69] as well. One clarification that should be provided at this point is that the acquisition of the technology components does not allow one to have a readily usable system. In fact, the usability of the system depends on the programming of its functioning through the MacroDroid app. This programming, albeit not particularly complex, requires a certain level of familiarity with technology issues. A similar point could be made regarding the implementation of the programs. Although not difficult, it requires staff to acquire a clear understanding of the procedural conditions and the technology arrangement. The authors of this study are willing to provide assistance to any context interested in adopting and using the technology system and programs reported.

Sixth, the fact that the system does not require adjustments from one session to the next and the evidence that the participants can be independent of any external assistance during the sessions can lead one to basic considerations about program applicability and sustainability [70-72]. For example, one might argue that (1) the participants involved in this study could continue to use the programs reported over time with relatively small staff time investment, and (2) the same programs could represent a realistic treatment opportunity for other people with extensive disabilities and limited access to stimulation and physical activity (ie, a treatment opportunity that their daily contexts might be able to afford in terms of time cost and technology requirements).

### Limitations and Future Research

While the results appear promising, some caution may be required in interpreting them given the limitations of the study. The most obvious limitations are the relatively small number of participants, the unavailability of maintenance and generalization data, and the absence of social validation of the programs and the technology system involved. An additional limitation may be the use of an experimental sensor (ie, the smartphone’s light sensor) to record the participants’ posture. With regard to the small number of participants, 2 points may need to be clarified. The first point is that the single-case research designs used with the participants involved in program 1 and the participants involved in program 2 can be considered adequate to support the internal validity of the data reported [44,45,73]. The second point is that new studies (ie, single-case replication studies and/or group-design studies) would be critically important to determine the external validity of the data and thus their level of representativeness [73-76].

To deal with the issues of maintenance and generalization, more comprehensive studies are needed. Those studies would have to (1) extend the data collection periods and include follow-up checks to verify whether the participants’ gains remain stable over time, (2) test the effects of the programs with different groups of people so as to verify whether those effects can be reproduced across participants, and (3) include different contexts in the evaluations (eg, homes, schools, and community and rehabilitation settings) to ascertain the applicability and effectiveness of those programs across settings as well as the sustainability of the programs within those settings [61,63,70,77,78].

To address the social validation question, new studies may need to gather the opinions of staff, family members, and service providers about the acceptability, relevance, and usability of the programs and the technology system on which they rely [79,80]. The process of gathering such opinions may involve 2 steps. The first step would consist of presenting all these personnel with videos of program sessions in which participants use the technology system. The second step would consist of presenting the same personnel with a series of questions concerning the programs and the technology (eg, their impact, social relevance, and applicability) [81-83]. The aforementioned process may be complemented by formal scales assessing the usability of technology (eg, the System Usability Scale) [84,85].

With regard to the recording of body posture, new studies may need to evaluate the usability and practicality of accelerometers, depth cameras, or infrared optical tracking sensors [86-88]. New studies may also consider ways of measuring (1) the impact of mild physical activity such as that adopted in the 2 programs of this study and (2) the level of purposefulness in the participants’ selection of the music pieces (ie, in their decision about the pressure sensor to activate at each music opportunity). To address the first issue, one might need to identify evaluation criteria and instruments more sophisticated than those presently available [9,51,58,64]. To address the second issue, one may need to associate highly preferred music pieces with one sensor and moderately preferred music pieces with the other sensor and verify whether the activation of the former sensor is more frequent than the activation of the latter sensor [13,89-91].

### Conclusions

The findings suggest that the programs assessed in this study were helpful in improving the situation of people with intellectual disability and visual or visual and motor impairments. All participants learned to activate music stimulation and to engage in recommended forms of physical activity. The participants involved in program 2 also showed an improvement in body posture. These findings provide new evidence of the possibility of developing technology-aided programs to improve the situation of participants with extensive disabilities. Notwithstanding this evidence, caution is required in making general statements and drawing conclusions based on these findings given the limitations of this study, which need to be addressed through new, carefully planned research efforts.

## Abbreviations

NAP: Nonoverlap of All Pairs

## Data Availability

The datasets generated or analyzed during this study are available in the OneDrive repository [92].
